# RACIAL AND ETHNIC DISPARITIES IN COGNITIVE DIFFICULTY AMONG OLDER ADULTS: EVIDENCE FROM NEW YORK CITY

**DOI:** 10.1093/geroni/igac059.1988

**Published:** 2022-12-20

**Authors:** Ethan Siu Leung Cheung, Jinyu Liu

**Affiliations:** Columbia University, New York, New York, United States; Columbia University, New York City, New York, United States

## Abstract

This study examined racial and ethnic disparities in cognitive difficulty among older adults in New York City (NYC). Also, we tested whether physical health, family structure, individual socioeconomic status (SES), and neighborhood SES explained the disparities. Based on community districts, individual-level data from the 2019 American Community Survey were merged with neighborhood data from NYC Community District Profile. A sample of 5,622 NYC residents aged 60 or older was included across 55 community districts. The outcome variable, cognitive difficulty, was measured by a binary variable in which respondents’ self-reported challenges with cognitive health (1=having challenge, 0=no). Racial and ethnic groups included Whites, Blacks, Latinos/Hispanics, and Asians. We used multilevel logistic regressions for analysis. Results show that Latinos/Hispanics had the highest odds of reporting cognitive difficulty across groups. Physical health, marital status, individual SES, and access to parks were significantly associated with cognitive difficulty. Physical health, family structure, and multilevel SES partially explained or influenced the racial and ethnic disparity in cognitive difficulty. However, such influence varied by race and ethnicity. Physical health and individual SES contributed to the disparities for Latinos/Hispanics and Blacks, compared to Whites. Neighborhood SES attenuated the disparity in cognitive difficulty between Latinos/Hispanics and Whites. Also, family structure uniquely explained the disparity for Blacks. No significant disparity was identified between Asians and Whites. This study shed light on the important roles of multilevel factors in predicting racial and ethnic disparities in cognitive difficulty. Findings provide direction for interventions to reduce racial and ethnic disparities in cognitive difficulty.

